# Prevalence and Factors Associated with Metabolic Syndrome among Brazilian Adult Population: National Health Survey - 2013

**DOI:** 10.5935/abc.20180072

**Published:** 2018-05

**Authors:** Elyssia Karine Nunes Mendonça Ramires, Risia Cristina Egito de Menezes, Giovana Longo-Silva, Taíse Gama dos Santos, Patrícia de Menezes Marinho, Jonas Augusto Cardoso da Silveira

**Affiliations:** Universidade Federal de Alagoas, Maceió, AL - Brazil

**Keywords:** Cardiovascular Diseases / mortality, Metabolic Syndrome / epidemiology, Epidemiology, Adult, Public Health Surveillance, Health Surveys

## Abstract

**Background:**

In Brazil, population-based researches analyzing prevalence and factors associated with metabolic syndrome (MS), a recognized predictor of cardiovascular diseases (CVD), and an important cause of disability and death in the country are scarce.

**Objective:**

To evaluate prevalence of MS and its associated factors in Brazilian population.

**Methods:**

Secondary analysis of the 2013 National Health Survey, a cross-sectional survey with national representativeness of Brazilian adult population (n = 59,402). MS was the outcome variable, defined from harmonization of cardiology international consensus as load ≥ 3 of the following components: self-reported diabetes and hypercholesterolemia, high blood pressure and high waist circumference. Analysis were stratified by sex and prevalence ratios, with their respective 99% confidence intervals (PR [CI 99%]) calculated by simple and multiple Poisson regression models.

**Results:**

MS prevalence was 8.9%, being significantly higher among women compared to men; in general, this pattern was maintained in relation to exposure variables studied. Additionally, less than 25% of population did not present any MS component. In final multiple models, sociodemographic, behavioral and comorbidity variables were associated with MS, however, while low schooling (1.46 [1.23-1.74], cerebrovascular accident (1.36 [1], 00] (1.28 [1.03-1.62]) were associated among women, chronic renal failure (1.85 [2.23-2.76]) was associated exclusively among men.

**Conclusion:**

We identified MS high prevalence in Brazilian population; on the other hand, factors associated with this condition were different depending on sex.

## Introduction

Changes that occurred in population socioeconomic and cultural patterns, as rapid urbanization and economic development consequence, resulted in significant changes in different population group life habits.^1^ This new society organization form, associated with alimentary transition and population aging, promoted transformations in the way that people get sick, increasing morbidity and mortality by Noncommunicable Diseases (NCD).^2^

Concerning specific cardiovascular diseases (CVD), risk factors concomitant presence such as hypertension, hypercholesterolemia, diabetes, insulin resistance and central fat deposition is associated with an approximately 2.5-fold increase in cardiovascular morbidity and mortality risk.^3^ This complex aggregate of CVD predisposing factors constitutes the condition defined as metabolic syndrome (MS).^4^

Worldwide MS recent estimate points to prevalence between 20-25% in adult population.^3^ In U.S. MS prevalence was 34.7% in 2011-2012. It was defined by harmonized criterion, which synthesizes other classification criteria developed by different organizations to define this condition.^5^ In Latin America cities, MS prevalence found between 2003 and 2005 was 21%, defined by National Cholesterol Education Program Expert Panel (NCEP-ATPIII) American criterion, presenting variation of 14% to 27%, according to studied territories.^6^ In Brazil, prevalence was even higher, varying around 30% among individuals aged 19 to 64 years in different country regions.^7^

In this situation, Brazilian government launched the Strategic Action Plan to Tackle Noncommunicable Diseases (NCD) in Brazil 2011-2022, which includes, among other actions, generating information and knowledge about health-disease process and its social determinants for health policies formulation in Brazil.^8^ In this sense, it was conceived the first National Health Survey (NHS) focused on risk factors surveillance and chronic diseases protection in Brazilian population.^9^

In this perspective, based on 2013 NHS data, this study objective was to estimate MS prevalence and its components on Brazilian population aged ≥ 18 years and its association with sociodemographic, behavioral and biological variables.

## Methods

### Design and sample of the study

NHS is a household-based cross-sectional survey with representativeness of Brazilian adult population conducted between August and December 2013. NHS was a population survey on health and its determinants, carried out in Brazil by the Brazilian Institute of Geography and Statistics (IBGE) allied to the Ministry of Health.^9^

The sampling process was organized by clusters in three stages, where the primary sampling unit (PSU) was composed by census sectors, secondary unit was households and tertiary unit, inhabitants 18 years old or older. Within each stage, participants were selected through simple random sampling. It was considered the corresponding households weight, the dweller selection probability, non-response adjustments by sex and calibration by population totals, by sex and estimated age classes, with all the dwellers weight. A detailed methodology applied description for NHS-2013 was previously published.^9^

To describe Brazilian population health conditions, NHS was constituted by thematic modules that addressed health and lifestyle individual perception, chronic diseases presence, as well as sociodemographic information. For the study purposes, individuals with data on the factors that make up MS were selected, that is, diabetes mellitus and hypercholesterolemia self-referred medical diagnosis, in addition to blood pressure (BP) values and waist circumference measurements (WC).

Among the 69.954 domiciles occupied with resident selected for NHS-2013 interview, 60,202 individuals 18 years old or older were interviewed, representing response rate of selected residents of 86%.^9^ We excluded 800 pregnant women from the total, generating a final sample composed of 59.402 individuals.

The National Commission of Ethics in Research (CONEP) approved the NHS project in June 2013, under Opinion Nº. 328,159. All the interviewees who agreed to participate in the investigation signed the term of free and informed consent.^9^

### Outcome variable

In this study, MS outcome variable was defined according to the international cardiology consensus harmonization proposal (IDF / NHLBI / AHA / WHF / IAS / IASO), characterized by the presence of three out of five metabolic risk factors.^4^

In our work, once NHS did not provide biochemical data, MS classification was made considering at least the presence of three of four situations available in database: 1) self-reported diabetes diagnosis; 2) BP values considered borderline for Systemic Hypertension diagnosis (HAS) (systolic ≥130 mmHg and/or diastolic ≥ 85 mmHg); 3) WC values above the cut-off point established as abdominal obesity threshold for South America population (men ≥ 90 cm and women ≥ 80 cm); and, 4) self-reported hypercholesterolemia diagnosis.

A trained team using a calibrated digital device evaluated BP. Individuals needed to be at rest and were oriented to empty their bladders, not smoke or drink during the 30-minute period prior to measurement and not perform any physical activity during the period of one hour before the measurement. BP measurements were made with individuals in sitting position, after having rested for at least five minutes. The individuals were instructed to stay relaxed and leaning against the back of the chair, not crossing their legs and leaving their left arm free of clothes and leaning on a table at the same level as their chest or heart. Three BP measurements were evaluated, with two minutes intervals between them. For the present study, the mean between the second and the third measures was used. In this technique, systolic and diastolic pressures were calculated by algorithms from the maximum oscillation point corresponding to average BP.^9^

As for anthropometric measurements, weight (kg), height (m) and WC (cm) were measured - having as reference for the perimeter the midpoint between last rib and iliac crest, being used, respectively, portable electronic scale, portable stadiometer, flexible and inelastic measuring tape with 0.1 cm precision. The procedures for anthropometry realization followed the same protocol used in the Brazilian Institute of Geography and Statistics (IBGE) 2008-2009 Family Budget Survey (FBS).^9^

### Independent variables

Independent variables selection was performed based on distal and proximal determinants conceptual model, developed to show sociodemographic, behavioral and comorbid multiple factors impact for MS on population health status.^10^

Sociodemographic factors used included: sex, age (18-59 years and ≥ 60 years), schooling (≤ 8 years and > 8 years), skin color (white / non-white), marital status (living with partner or not), macro-region (South / Southeast /Center-West and North/Northeast). Macro regions were dichotomized in order to contrast Brazilian development poles, with the South, Southeast and Central West regions being the most developed. Behavioral variables were self-perceived health, considering the combination of "very good" and "good" responses, defining as reference "regular", "poor" and "very poor" responses.

For variable physical activity construction (PA), the following information were considered regarding free time and volume, effort duration and intensity for physical activity, using three months preceding the day of the interview as reference period for PA questions in the questionnaire. Effort intensity was obtained by conversion of physical exercise type or sport reported in vigorous or moderate PA as determined by the Physical Activity Compendium (PAC).^11^ Effort duration in each session was expressed in minutes and divided into three groups: < 19; 20 to 29; > 30 minutes. Weekly frequency was determined by PA practice day number per week and, for analysis purpose, was divided into 0 to 2 days, 3 to 4 days and > 5 days per week.

Thus, PA variable was initially grouped into three categories, according to World Health Organization recommendation: active (individual that reaches or exceeds moderate physical activity for 150 minutes or vigorous physical activity for 75 minutes per week in at least 10 minutes sessions); inactive (which refuses to practice PA at leisure) and insufficient assets (when performing PA below recommendation).^11^ Finally, it was decided to combine inactive and insufficient assets categories, transforming them into dichotomous variable (active/inactive).

Variables corresponding to comorbidities analyzed here were previous self-reported cerebral vascular accident medical diagnoses (CVA), chronic renal failure (CRF), depression and other cardiovascular diseases (CVD). For the latter, previous CVD diagnoses reports were considered, such as: infarction, angina, heart failure, among others. Overweight was identified according to body mass index cut-off points (BMI). In individuals aged between 18 and 59 years, values ≥ 25 kg/m^2^ were considered overweight.^11^ For those aged 60 years or older, values > 27 kg/m^2^ were considered as overweight.^12^

### Statistical analysis

Statistical analysis were processed with Stata software version 13.0 (Stata Corp., College Station, USA), using survey commands, whose analysis procedures take into account sampling design and weight.^13^

The comparison between MS prevalence, for each comorbidity and for disease burden was based on their respective confidence intervals of 99% (CI 99%). Prevalence ratios (PR) with their respective CI 99%, were calculated by simple and multiple Poisson regression models.^14^

Statistical modeling process was conducted using determinants conceptual model to MS,^10^ applying hierarchical approach in analysis and using stepwise forward method for variables introduction, considering as eligible those with p < 0.20 (univariate analysis); variables in which the CI 99% did not include "1" or contributed to model adjustment remained in the model.

Associations between MS and potential associated factors were introduced according to sociodemographic, behavioral and comorbidity factors, analyzed by three multiple models. At distal level of analysis (Model 1), the sociodemographic variables age, schooling, skin color, conjugal situation and housing region were considered; for Model 2 composition we used, behavioral variables physical activity and health self-perception adjusted by Model 1; In model 3, variables referring to proximal determinants (comorbidities) were introduced and their effects being adjusted by model 2. It is emphasized that once variables set was defined in a hierarchically superior model, it did not suffer any alteration in others levels of analysis.

Justification for preserving variables in each model was based on result importance for MS occurrence understanding and effect magnitude, as well as its variability, represented here by CI 99%.^14^ In addition, analysis were stratified by sex, considering that in descriptive analysis, MS showed to affect in different way male and female population, which may reflect different association factors between the groups.

## Results

Sociodemographic, behavioral and comorbidities characteristics of 59.402 individuals over 18 years are described in Table 1 according to MS absence or presence. Physically inactive individual high frequency was identified (98.1%) and 53.8% who were overweight. Self-perception predominant report of very good or good health was observed (65.9%) and low schooling significant frequency (39.1%) among individuals.

**Table 1:** Sociodemographic, behavioral and comorbidity characteristics of adults and elderly - National Health Survey (NHS), Brazil, 2013

Variables	MS*					
	Total*		Male		Female	
	N = 59.402†		N = 25.920†		N = 33.482†	
	%*	CI 99%	%*	CI 99%	%*	CI 99%
**Age**						
18-59 years	81,8	80,9 - 82,5	83,3	82,4 - 84,1	80,4	79,6 - 81,1
≥ 60 years	18,2	17,4 - 19,0	16,7	15,9 - 17,5	19,6	18,8 - 20,9
**Education**						
> 8 years	60,9	59,7 - 62,0	60,2	58,9 - 61,4	61,5	60,5 - 62,5
≤ 8 years	39,1	37,9 - 40,2	39,8	38,6 - 41,0	38,5	37,4 - 39,5
**Skin color**						
White	47,4	46,4 - 48,5	46,8	45,7 - 47,9	48,1	47,0 - 49,1
Non-white §	52,5	51,5 - 53,6	53,2	52,1 - 54,3	51,9	50,9 - 53,0
**Conjugal situation**						
Do not live with a partner	55,7	54,6 - 56,7	53,4	52,2 - 54,6	57,8	56,8 - 58,8
Live with a partner	44,3	43,2 - 45,3	46,6	45,4 - 47,8	42,2	41,2 - 43,2
**Housing region**						
NE/N	34,0	33,3 - 34,7	34,2	33,4 - 35,1	33,8	32,9 - 34,6
SE/S/CW	66,0	65,3 - 66,7	65,8	64,9 - 66,6	66,2	65,4 - 67,0
**Physical activity**						
Active	1,9	1,6 - 2,2	2,2	1,9 - 2,5	1,6	1,3 - 1,9
Inactive	98,1	97,8 - 98,4	97,8	97,5 - 98,1	98,4	98,0 - 98,6
**Health self-perception**						
Very good/good	65,9	65,0 - 66,9	70,3	69,3 - 71,2	62,1	61,1 - 63,1
Regular - Very bad	34,1	33,0 - 34,9	29,7	28,8 - 30,7	37,9	36,9 - 38,9
**Overweight**						
No	46,2	45,1 - 47,3	47,5	46,4 - 48,7	45,0	43,9 - 45,9
Yes	53,8	52,7 - 54,9	52,5	51,3 - 53,6	55,0	54,0 - 56,0
**Another CVD ^‡^**						
No	95,8	95,3 - 96,2	96,1	95,6 - 96,5	95,5	95,1 - 95,9
Yes	4,2	3,8 - 4,7	3,9	3,5 - 4,4	4,5	4,1 - 4,9
**Chronic renal failure**						
No	98,6	98,3 - 98,8	98,6	98,4 - 98,9	98,5	98,2 - 98,7
Yes	1,4	1,2 - 1,7	1,4	1,1 - 1,6	1,5	1,3 - 1,7
**Stroke**						
No	98,5	98,2 - 98,7	98,4	98,1 - 98,6	98,5	98,3 - 98,7
Yes	1,5	1,3 - 1,8	1,6	1,4 - 1,9	1,5	1,3 - 1,7
**Depression**						
No	92,3	91,7 - 92,9	96,1	95,6 - 96,5	88,9	88,3 - 89,6
Yes	7,7	7,1 - 8,2	3,9	3,5 - 4,4	11,1	10,4 - 11,7

MS: metabolic syndrome; CI 99%: confidence interval 99%; N: norte; NE: northeast; SE: southeast; S: south; CW: central-west; CVD: cardiovascular disease;

(*) Generated considering the sample weight;

(†) Number of individuals in the database;

(§) Yellow, indigenous, brown, black

(‡) infarction, angina, heart failure or other.

Table 2 shows MS prevalence, comorbidities and MS components burden in Brazilian population. Abdominal obesity was the factor with highest prevalence in this study (65.2%, CI 99% 64.4-65.9), followed by high BP (40.7%, CI 99% 39.6-41.7). It was observed that in all comorbidities, women presented the most expressive results, with high BP (46.9%, CI 99% 45.5-48.3) the only condition in which men showed a higher prevalence. In components sum, it is observed that only 1/4 of population did not present any of studied changes (23.8% [CI 99% 22.9-24.7]), while 38.1% (CI 99% 37.2-39.0) of participants already presented at least one MS component and 29.2% (CI 99% 28.3-30.1) coexisted with two considered factors. MS condition was estimated at 8.9% (CI 99% 8.4-9.5) of Brazilian population, with women proportion in this condition (10.3% [CI 99% 9.6-11.2]) statistically surpassing what was observed in the male population (7.5% [CI 99%, 6.7-8.3]).

**Table 2:** Prevalence (%) of comorbidities and burden of diseases (risk factors for MS), by sex, of adults and elderly. National Health Survey (NHS), Brazil, 2013

Disease, burden and MS			Sex			
	Total N = 59.402		Male N = 25.920		Female N = 33.482	
	%*	CI 99%	% *	CI 99%	% *	CI 99%
Diabetes	7,1	6,6 - 7,6	6,3	5,6 - 7,2	7,8	7,1 - 8,5
High BP	40,7	39,6 - 41,7	46,9	45,5 - 48,3	34,9	33,7 - 36,3
Hypercholesterolemia	14,7	14,0 - 15,5	11,9	10,9 - 13,1	16,9	16,0 - 17,9
High WC	65,2	64,4 - 65,9	55,6	54,0 - 57,1	73,9	72,7 - 75,0
**Disease burden**						
0	23,8	22,9 - 24,7	28,0	26,7 - 29,4	19,9	18,8 - 21,1
1	38,1	37,2 - 39,0	34,8	33,5 - 36,2	41,0	39,8 - 42,3
2	29,2	28,3 - 30,1	29,7	28,4 - 31,0	28,7	27,6 - 29,9
3	7,5	7,1 - 8,1	6,5	5,8 - 7,3	8,5	7,8 - 9,3
4	1,4	0,9 - 1,2	1,0	0,7 - 1,3	1,8	1,5 - 2,2
MS Condition †	8,9	8,4 - 9,5	7,5	6,7 - 8,3	10,3	9,6 - 11,2

MS: metabolic syndrome; N: number of individuals in the database; CI 99%: confidence interval 99%; BP: blood pressure; WC: waist circumference;

(*) Generated considering the sample weight;

(†) MS Condition, sum of disease burden ≥ 3 factors.

Table 3 presents MS prevalence according to studied exposure variables. There are greater aggravation prevalence among older individuals (≥ 60 years), lower schooling time (≤ 8 years) and living with a partner. MS was greater among individuals residing in SE/S/CW regions, physically inactive, with overweight and who considered their health precarious. Regarding comorbidities, in general, MS higher prevalence were found among individuals who reported prior CKD diagnosis, stroke and other cardiovascular diseases, in relation to those who said they did not have the disease. In addition, we identified that, regardless of characteristic or condition considered as risk, MS prevalence was always higher among women.

**Table 3:** MS Prevalence according to exposure variables studied - National Health Survey (NHS), Brazil, 2013

Variables	MS*					
	Total		Male		Female	
	N = 59.402		N = 25.920		N = 33.482	
	p (%)*	CI 99%	p (%)*	CI 99%	p (%)*	CI 99%
**Age**						
18-59 years	5,8	5,3 - 6,3	5,4	4,7 - 6,3	6,1	5,5 - 6,8
≥ 60 years	23,2	21,4 - 25,1	17,5	14,7 - 20,8	27,6	25,0 - 30,3
**Education**						
> 8 years	6,3	5,7 - 6,9	6,8	5,8 - 7,8	5,8	5,1 - 6,6
≤ 8 years	13,1	12,1 - 14,2	8,4	7,1 - 9,9	17,6	15,9 - 19,3
**Skin color**						
White	9,7	8,9 - 10,6	8,9	7,7 - 10,5	10,4	9,3 - 11,6
Non-white†	8,3	7,5 - 9,0	6,1	5,2 - 7,1	10,3	9,2 - 11,4
**Conjugal situation**						
Do not live with a partner	6,9	6,4 - 7,6	4,6	3,8 - 5,6	8,9	8,1 - 9,8
Live with a partner	11,5	10,5 - 12,5	10,7	9,3 - 12,2	12,3	10,9 - 13,7
**Housing region**						
NE/N	7,3	6,6 - 8,0	5,3	4,4 - 6,2	9,1	8,0 - 10,3
SE/S/CW	9,8	9,1 - 10,6	8,6	7,5 - 9,8	10,9	9,9 - 12,1
**Physical activity**						
Active	1,8	0,7 - 4,9	2,5	0,7 - 8,6	1,0	0,3 - 3,4
Inactive	9,1	8,6 - 9,7	7,6	6,8 - 8,4	10,5	9,7 - 11,3
**Health self-perception**						
Very good/good	4,8	4,3 - 5,3	4,5	3,8 - 5,4	5,0	4,4, 5,7
Regular - Very bad	17,1	15,9 - 18,3	14,4	12,5 - 16,5	19,0	17,4 - 20,7
**Overweight**						
No	4,8	4,3 - 5,5	3,4	2,6 - 4,4	6,2	5,4 - 7,2
Yes	12,5	11,7 - 13,4	11,1	9,9 - 12,5	13,7	12,5 - 14,9
**Another CVD ^‡^**						
No	8,3	7,7 - 8,8	6,9	6,1 - 7,7	9,5	8,8 - 10,3
Yes	24,9	20,9 - 29,4	21,8	15,9 - 29,2	27,3	22,2 - 33,2
**Chronic renal failure**						
No	8,8	8,2 - 9,3	7,2	6,4 - 8,1	10,2	9,4 - 11,0
Yes	21,9	16,6 - 28,3	25,7	17,3 - 36,3	18,8	12,8 - 26,8
**Stroke**						
No	8,7	8,1 - 9,2	7,2	6,4 - 8,1	10,0	9,3 - 10,8
Yes	27,0	20,6 - 34,5	22,7	14,2 - 34,2	31,4	22,6 - 41,7
**Depression**						
No	8,3	7,7 - 8,8	7,1	6,3 - 7,8	9,4	8,6 - 10,3
Yes	17,1	14,6 - 19,9	15,6	10,7 - 22,3	17,6	14,7 - 20,9

MS: metabolic syndrome; CI 99%: confidence interval 99%; N: norte; NE: northeast; SE: southeast; S: south; CW: central-west; CVD: cardiovascular disease;

(*) Generated considering the sample weight;

(†) Number of individuals in the database;

(§)Yellow, indigenous, brown, black

(‡) infarction, angina, heart failure or other.

Results referring to factors associated with MS in hierarchical modeling process (hypothetical-causal model), different for men and women, are available in tables 4 and 5. In female population final model, we identified that MS probability was higher among individuals in the following situations: age ≥ 60 years (PR 3.20 [CI 99% 2.76-3.72]), education ≤ 8 (PR 1.46 [CI 99% 1, 23-1,74]), living with partner (PR 1.27 [CI 99% 1,11-1,45]), residing in SE/S/CW regions (PR 1.18 [CI 99% 1, 02-1.38]), regular to very bad health self-perception (PR 2.35 [CI 99% 1.99-2.78]), stroke (PR 1, (CI 99% 1.00-1.86), other CSD (PR 1.29 [CI 99% 1.03-1.62]), overweight (PR 2.09 [CI 99% 1, 79-2.42]) and depression (PR 1, 31 [CI 99% 1.07-1.59]) (Table 4).

**Table 4:** Bivariate analysis and multivariable models for factors associated with metabolic syndrome among Brazilian women according to hierarchical levels of exposure variables studied - National Health Survey (NHS), Brazil, 2013

Variables	Bivariate analysis		Model 1		Model 2		Model 3	
	PR	CI 99%	PR	CI 99%	PR	CI 99%	PR	CI 99%
**Age**								
18-59 years	1		1		1		1	
≥60 years	4,49	3,90- 5,18	3,44	2,95- 4,01	2,99	2,56- 3,48	3,20	2,76- 3,72
Education								
>8 years	1		1		1		1	
≤ 8 years	3,01	2,55- 3,55	1,98	1,67- 2,34	1,54	1,29- 1,83	1,46	1,23- 1,74
Conjugal situation								
Do not live with a partner	1				1		1	
Live with a partner	1,37	1,18- 1,58	1,38	1,21- 1,58	1,33	1,17- 1,52	1,27	1,11- 1,45
Housing region								
NE/N	1		1		1		1	
SE/S/CW	1,20	1,02- 1,41	1,18	1,01- 1,36	1,30	1,12- 1,52	1,18	1,02- 1,38
Skin color								
White	1							
Non-white*	0,98	0,84- 1,15						
Physical activity								
Active	1							
Inactive	10,06	3,08- 32,84						
Health self-perception								
Very good/good	1				1		1	
Regular - Very bad	3,76	3,23- 4,38			2,65	2,24- 3,14	2,35	1,99- 2,78
Stroke								
No	1						1	
Yes	3,13	2,29- 4,27					1,36	1,00- 1,86
Another CVD†								
No	1						1	
Yes	2,86	2,32- 3,51					1,29	1,03- 1,62
Overweight								
No	1						1	
Yes	2,19	1,85- 5,59					2,09	1,79- 2,42
Depression								
No	1						1	
Yes	1,86	1,54- 2,26					1,31	1,07- 1,59
Chronic renal failure								
No	1							
Yes	1,84	1,26- 2,69						

PR: prevalence ratio; CI 99%: confidence interval 99%; N: norte; NE: northeast; SE: southeast; S: south; CW: central-west; CVD: cardiovascular disease;

(*) Yellow, indigenous, brown, black

(‡) infarction, angina, heart failure or other.

**Table 5:** Bivariate analysis and multivariable models for factors associated with metabolic syndrome among Brazilian men according to hierarchical levels of exposure variables studied - National Health Survey (NHS), Brazil,2013

Variables	Bivariate analysis		Model 1		Model 2		Model 3	
	PR	CI 99%	PR	CI 99%	PR	CI 99%	PR	CI 99%
**Age**								
18-59 years	1		1		1		1	
≥ 60 years	3,23	2,55- 4,08	2,74	2,17- 3,46	2,07	1,61- 2,67	2,60	2,04- 3,31
**Education**								
> 8 years	1							
≤ 8 years	1,24	0,99- 1,54						
**Conjugal situation**								
Do not live with a partner	1		1		1		1	
Live with a partner	2,30	1,82- 2,92	1,81	1,43- 2,30	1,74	1,37- 2,20	1,48	1,17- 1,88
**Housing region**								
NE/N	1		1		1		1	
SE/S/CW	1,63	1,31- 2,02	1,49	1,20- 1,85	1,74	1,40- 2,14	1,57	1,28- 1,94
**Skin color**								
White	1							
Non-white†	0,67	0,54- 0,84						
**Physical activity**								
Active	1							
Inactive	3,02	0,84- 10,90						
**Health self-perception**								
Very good/good	1				1		1	
Regular - Very bad	3,17	2,51- 4-01			2,72	2,12- 3,50	2,59	2,01- 3,33
**Stroke**								
No	1							
Yes	3,15	1,99- 4,98						
**Another CVD‡**								
No	1							
Yes	3,17	2,29- 4,39						
**Overweight**								
No	1						1	
Yes	3,27	2,45- 4,37					3,58	2,73- 4,70
**Depression**								
No	1						1	
Yes	2,19	1,49- 3,23					1,41	0,98- 2,02
**Chronic renal failure**								
No	1						1	
Yes	3,57	2,41- 5,28					1,85	1,23- 2,76

PR: prevalence ratio; CI 99%: confidence interval 99%; N: norte; NE: northeast; SE: southeast; S: south; CW: central-west; CVD: cardiovascular disease;

(*) Yellow, indigenous, brown, black

(‡) infarction, angina, heart failure or other.

Regarding male population, final model did not include variables as schooling, skin color, other CVD and CVA, age remaining in ≥60 years (PR 2.60 [CI 99% 2.04-3.31]), living with partner (PR 1.48 [CI 99% 1.17-1.88]), residing in SE/S/CW regions (PR 1.57 [CI 99% 1.28-1.94]), have worse ("regular to very bad") self-referred health (RP 2.59 [IC99% 2.01-3.33]) and present IRC (RP 1.85 [IC99% 2.23-2, 76]), overweight (RR 3.58 [IC99% 2.73-4.70]) and depression (RP 1.41 [IC99% 0.98-2.02]) (Table 5).

Regarding the physical activity variable, in order not to compromise analysis, we chose not to include it in the model, given the low prevalence of physically active individuals (1.8%), which would result in inaccurate estimates, due to large standard error (Table 4 and 5). In addition, considering the referential adopted, such comparisons become unnecessary, since almost entire Brazilian population is characterized as physically inactive (98.1%) (Table 1).

## Discussion

MS is a multidimensional phenomenon determined by an interaction factor set that affects people quality of life.^3^ Despite the existence of studies on chronic diseases and their risk factors in Brazilian population,^15^ as the Adult Health Longitudinal Study, we do not yet have MS prevalence national representativeness in the country. Thus, the present study evaluated for the first time the factors associated with MS in a Brazilian population representative sample aged over 18 years, representing a milestone in the MS investigation in Brazil, collaborating in construction of evidence capable of directing resolute strategies for prevention and control of this problem.

We identified that approximately 9% Brazilian population presented MS status, according to consensuses harmonization definition.^4^ Nevertheless, our study reveals worrying data, accounting that only 23.8% of population does not present any MS component, and that 67.3% have between one and two components to characterize this outcome, which demonstrates high number of individuals under developing MS risk.

Considering sex, highest MS occurrence was found among female population (10.3%, CI 99% 9.6-11.2), fact that has been widely recorded in scientific literature, ^16^^,^^17^ especially among those with age > 59 years, which can be explained by hormonal changes that occurred after menopause.^17^ At this moment of life, there is tendency to accumulate abdominal fat and also to increase density of LDL particles circulating in bloodstream that become more atherogenic, known condition associated to increased risk of CVD.^18^ It is also possible to highlight the high number of morbidity found among women in this study, who presented higher prevalence of diabetes, hypercholesterolemia and abdominal obesity in relation to masculine sex.

Considerable differentiation in MS abnormalities prevalence and combinations between the sexes suggests different physiopathology between men and women^19^ possibly explained by sex hormones different levels that influence metabolism regulatory mechanisms.^20^ The greater androgenic activity found among men as well as postmenopausal estrogen levels reduction among women are conditions that favor an increase in visceral abdominal fat and in bloodstream lipids concentration, which is correlated with insulin resistance, hypertension, and increased cardiovascular risk.^20^

Among MS components, high WC results (65.2%, CI 99% 64.4-65.9) are highlighted in the study population. Abdominal obesity plays an important role in MS,^21^^,^^22^ because it is associated with a metabolic disorder capable of damaging the artery wall, leading to vasoconstriction deregulation, inflammatory cascades activation, and adipokines effects elevation, considered to be factors that induce CVD.^22^ Cohort study conducted by Lee et al.^22^ showed that the additional 500 cm^3^ volume increase of subcutaneous and visceral fat is associated with MS incidence and risk factors aggravation for CVD.^22^ A obese individuals cohort study conducted in Italy showed that abdominal obesity contributed to MS prevalence in obese women, but not in men.^19^

In our study, a possible explanation for elevated WC high prevalence can be derived from the lower cut-off points established by ethnicity standardization for abdominal obesity (≥ 90 cm for men and ≥ 80 cm for women),^4^ when compared with limit values NCEP-ATPIII (102 cm for men and 88 cm for women).^23^ Therefore, this high and worrisome abdominal obesity prevalence in Brazilian population reflects in the increased risk of developing some cardiovascular disease^22^ and consequent risk for increased morbidity and mortality and impact on the health system.^2^

Positive relationship between MS prevalence and increase in age, found here, has been widely disseminated, especially due to differences attributed to sex,^17^ not only due to cumulative effect, reflecting the exposure time to risk factors related to inappropriate eating behaviors and unhealthy lifestyles, as well as the biological factors contribution in both sexes, due to direct relationship with testosterone/estrogen levels balance, specifically among women.^20^ MS has been slightly more diagnosed among men with less than fifty years of age, fact that is reversed after fifty years, affecting female population with greater magnitude.^17^

From sociodemographic point of view, this study showed that living in SE/S/CW regions was presented as factor associated with MS, which can be explained, in part, by these regions concentrating the country main urban centers, contributing to lifestyles promotion characterized by unhealthy eating habits and physical exercises low frequency, which have as consequence, increased risk of obesity, type 2 DM, arterial hypertension, CVD and MS.^5^

In a systematic review carried out among South American countries, this hypothesis was confirmed by showing that western eating habits and lifestyle pattern were found in greater proportion in large urban areas where chronic no communicable diseases were related to these behaviors.^24^ In addition, through an investigation conducted in Brazil, it was evident that there was a high calories consumption in the capitals located in the South, Southeast and Center-West regions, while in the capitals in the North and the Northeast a consumption below average was observed among the population.^25^

It is important to point out that in these large urban centers regions, society is increasingly induced by food offers messages, diets and western behaviors.^26^ In recent years, in Brazil, metropolitan regions have shown reduction in household consumption of polished rice, beans, cereals and legumes (down 60%, 49% and 25%, respectively), with a concomitant continuous consumption increase of foods such as: beverages and infusions (22% and 24%), prepared foods and industrial mixtures (67%), highlighting 490% increase in the amount of non-alcoholic beverage purchased between 1974 and 2009, evidencing a change in food behavior that is not always favoring healthy choices in developed regions.^27^

Our results showed that education lowest level was associated with MS prevalence in women, but this association was not evident among male population. This finding is consistent with the international literature, as observed in a study conducted in China, where educational level was also inversely related to MS prevalence strictly among women.^28^ In Korea, distinctly, it was identified that men with less schooling had lower odds ratios (OR) for both SM (OR 0.76, 95% CI 0.60-0.96), as well as for two of its isolated components: high WC (OR 0.73, 95% CI 0, 60-0.91) and low HDL cholesterol (OR 0.73, 95% CI 0.59-0.91), compared to men with higher education.^29^ In this study, a higher MS prevalence was observed among the socially disadvantaged women who had lower education level, unemployed, lower income level and who performed manual labor, as highlighted in our results in educational level terms.^29^

However, an antagonistic result was observed in high educational level male population, where cardiovascular risk factors distribution was more observed in this population stratum. This may be a reflection of the socioeconomic environment, which increases MS risk in one of the genders more specifically.^30^

In relation to conjugal situation variable, it is not clear how civil status contributes to MS. In the present study, the situation of living with a partner was associated to MS and to a greater extent in male population. This finding was also evident among Australians, where women who married gained more weight compared to unmarried women, after adjusting for potential confounding factors.^31^ Similarly, in the study by Averett et al.,^32^ marriage was associated with an increased risk of overweight/obesity, both for men and women.^32^

Ortega et al.,^33^ identified that sex affects the relationship between obesity, cardiometabolic risk factors and marital status due to changes in behavioral factors.^33^ Although most studies have reported that married people became more sedentary,^34^ with direct reflection in overweight and impact on MS comorbidities, collaborating with the results found in this work, other studies show different results, attributing to marital relationship type the MS outcome.^26^^,^^35^ In this case, it is considered that positive conjugal relationships can protect from stressful situations, providing both material and support benefits, while negative relationships or lack of relationship can increase exposure to conflicts, with consequent stress level elevation,^35^ factor recognized as associated to MS.^10^

Regarding behavioral aspects, we identified that "regular to very bad" health negative self-perception was important factor associated to MS. Health self-perception is global indicator in which the person considers, in addition to possible diseases, the impact they generate on physical, social and mental well-being.^32^ In Spain, by contrasting our results, in a multicenter study performed with DM and/or MS carriers recently diagnosed, 42.2% of the individuals believed to have good or excellent health, representing little awareness of the cardiovascular risk they presented.^36^ In this work, the association of negative self-perception "regular to very bad "with MS draws attention, because it may reflect that the studied population was aware of their health condition, but remains within the risk range for metabolic complications development, assuming that there may be other factors that prevent them from leaving this condition and deserve to be better investigated.

Regarding MS prevalence and association with comorbidities (CVD, stroke and depression), all were more frequent among females compared to males, with CRF exception. Association between MS and CRF found exclusively among men can be partly justified by hypertension high prevalence installed in this group.^19^ In recent decades, it has become increasingly evident that hypertensive patients prognosis is strongly affected by renal impairment especially in terms of mortality and cardiovascular events.^37^ In relation to the greater number of comorbidities associated to MS among women in the study, it can be explained in part by the low demand of men for health services, which generates an underdiagnosis.^2^

CVDs are the leading cause of death in Brazil,^2^ and should be a public health priority, through policies for their prevention and control. Association between DCV and MS was demonstrated in a Danish cohort, in which older women with MS presented risk of 1.7 (95% CI 1.44-2.05) for CVD development.^38^ Correlation can also be explained by Salas et al.,^16^ who showed that obese adults, mainly women, are particularly at risk of developing MS, with significant implications for their health, especially CVD and diabetes. These results highlight the weight loss importance to reduce morbidities associated to MS.^16^

Another disease condition associated with MS was depression, with higher prevalence found among women. Similarly, study conducted in Korea with middle-aged individuals aged 40-64 years found high prevalence among women with 11.7%, compared to 4.1% in men.^39^ These findings may be similar to what was found in disease prospective cohort among the Dutch population, where depression was significantly associated with higher WC and triglyceride levels during a 6-year follow-up.^40^ In this context these results may imply that older age may be associated with an increased response to stress and cortisol levels, more frequently among women than men.^39^

Methodological pattern reinforced presented results robustness; however, some limitations must be addressed. Up to the time of submission of this study, IBGE had not published HDL laboratory data, which led us to characterize MS in the occurrence of three of the four - and not five - components available in the database.

Another point refers to the use of self-reported medical diagnoses. The investigated population answered if "*some doctor already gave him the diagnosis of diabetes*?" Or "(...) *hypercholesterolemia*?", which reduces classification bias, because they were considered present when there was a positive response to the previous medical diagnosis of these diseases; On the other hand, given the issues of underdiagnoses, there is a real possibility of the presence of individuals who did not know their health condition at the time of the interview. In any case, even in these situations, we observed that MS high prevalence in our study is consistent with the literature,^7^^,^^10^^,^^16^ which warns of the possibility that this prevalence may be underestimated, implying a negative prognosis for Brazilian population aged ≥18 years.

Another limitation of this study is its cross-sectional design, which is why socioeconomic, behavioral and comorbidities factors analyzed here can not be unequivocally considered causal for MS. Especially in reference to comorbidities, it is important to highlight reverse causality role in identified associations, since clinical course start for CVD, stroke, depression and CRI would be in MS, despite the fact that delineation does not allow to affirm that this fact occurred in the studied population. However, use of data with national representativeness and conceptual model to base not only variables selection, but also analytical strategy, allows relevant information production for Brazilian population health conditions diagnosis, in which refers to MS, aligned with national public health priorities.

## Conclusion

We identified high prevalence of MS in adult and elderly population in Brazil, being associated with sociodemographic variables (age, schooling, conjugal status and housing region), behavioral (self-perception of health) and comorbidities (stroke, CVD, weight, depression and CRF) differently between the sexes.

Finally, the relevance of the burden of each MS component, reiterates MS as a clinical and epidemiological tool in the identification of individuals and population groups with greater vulnerability to CVD occurrence and as a guideline to cost-effective interventions on factors presented. Thus, our results suggest the need to strengthen public policies for health promotion in order to encourage the adoption of healthy behaviors; otherwise, it will be unlikely to fulfill the goals set forth in the Strategic Action Plan to Tackle Noncommunicable Diseases (NCD) in Brazil 2011-2022.
